# Comparison of the TruView PCD video laryngoscope and macintosh laryngoscope for pediatric tracheal intubation by novice paramedics: a randomized crossover simulation trial

**DOI:** 10.1007/s00431-015-2538-0

**Published:** 2015-04-18

**Authors:** Łukasz Szarpak, Łukasz Czyżewski, Andrzej Kurowski, Zenon Truszewski

**Affiliations:** Department of Emergency Medicine, Medical University of Warsaw, Lindleya 4 Street 02-005, Warsaw, Poland; Department of Anesthesiology, Institute of Cardiology, Warsaw, Poland; Department of Nephrologic Nursing, Medical University of Warsaw, Warsaw, Poland

**Keywords:** Cardiopulmonary resuscitation, TruView, Macintosh, Laryngoscope, Paramedic, Simulation

## Abstract

The aim of the present study was to evaluate whether the TruView video laryngoscope (TruView) facilitates pediatric endotracheal intubation (ETI) more quickly and safely than conventional Macintosh laryngoscope (MAC) in three manikin-based airway scenarios. This was a randomized crossover manikin study including 120 novice paramedics. The participants performed tracheal intubations using both TruView and MAC on a pediatric manikin in a control scenario (A), chest compression scenario (B), and chest compression cervical stabilization scenario (C). The sequence of scenarios was randomized. The primary outcome was time to intubation. Secondary outcomes were overall success rates, incidence of dental trauma, and ease of intubation. All intubation attempts were assessed by a trained assistant. The overall success rate was significantly higher with the TruView compared than the MAC in scenario B (100 vs. 81.7 %; *p* = 0.011) and scenario C (100 vs. 68.3 %; *p* < 0.001). The intubation time was significantly lower with the TruView than the MAC (18.5 vs. 24.3 s, *p* = 0.017, for scenario A; 21.6 vs. 25.7 s, *p* = 0.023, for scenario B; and 28.9 vs. 45.4 s, *p* < 0.001, for scenario C). Glottic view quality was better with TruView than the MAC in all scenarios, *p* < 0.001.

*Conclusions*: The TruView offers better intubation conditions than the MAC on a pediatric manikin in the control scenario, chest compression scenario, and chest compression scenario with cervical stabilization scenario. The TruView may be used to elevate the epiglottis for orotracheal intubation. Further clinical studies are necessary to confirm these initial positive findings.

**Trial Registration**: clinicaltrials.gov Identifier: NCT02289872.

**Table:** 

**What is Known:** •*Prehospital pediatric intubation using a standard laryngoscope is varied and ranges from 63.4 to 82 %.*
**What is New:** •*This is the first study showing efficiency of pediatric endotracheal intubation using the TruView PCD by paramedics in tree simulation scenarios.* •*TruView PCD offers better pediatric intubation conditions than the Macintosh laryngoscope.*

## Introduction

Since the invention of the Macintosh and Miller laryngoscope blades in the 1940s, direct laryngoscopy (DL) has been considered as the “gold standard” of endotracheal intubation (ETI). However, according to scientific studies, the effectiveness of the ETI on children performed by paramedics using a standard laryngoscope in pre-hospital care is insufficient and ranges from 63.4 to 77 % [8, 9]. In the light of the fact that one in four children requiring ETI and adequate ventilation is not intubated, or the endotracheal tube is incorrectly inserted [8, 9]. The ETI is considered the standard for securing the airway of severely ill or injured patients [1, 33]. According to current guidelines on the treatment of severely injured patients [2], emergency ETI should be performed immediately on all patients with a Glasgow Coma Scale (GCS) < 9 by emergency medical service (EMS) providers. Also, the 2010 European Resuscitation Council (ERC) [1] and American Heart Association (AHA) resuscitation guidelines [11] emphasize ETI as an airway management method during cardiopulmonary resuscitation (CPR). The ERC guidelines for CPR recommend that chest compressions are continued and interruptions are minimized during CPR, and ETI during resuscitation should be performed quickly and efficiently by an experienced operator, while interruptions to chest compressions should be avoided where possible.

Securing the airway using a tracheal tube brings many benefits. Firstly, it allows the use of asynchronous resuscitation while eliminating chest compression interruption for performing rescue breaths [1, 33]. It is also possible to use positive end-expiratory pressure (PEEP), as well as the constant measurement of the concentration of carbon dioxide in exhaled air [30]. Opinions on the intubation of children by paramedics in the prehospital care are varied [10, 22, 38]. However, paramedic working in EMS in Poland must have the ability to intubation, both children and adults. Philip Ragg noticed the benefits of using video laryngoscopy during child intubation and suggested an extension to the algorithm of Difficult Airway Society (DAS) on the use of video laryngoscopes in “Plan A” [33]. Several studies indicate that the use of video laryngoscopy in emergency situations can increase the effectiveness of intubation [12, 14, 36].

The aim of the study was to compare time and success rates of the TruView PCD video laryngoscope and the Macintosh laryngoscope (MAC) for pediatric emergency intubation with three airway scenarios on a standardized manikin model.

## Methods

This open, prospective, randomized, crossover manikin study was approved by the Program Committee of the International Institute of Rescue Research and Education (Head: Dr. A. Kurowski, 10.2014.05.15 on September 3rd, 2014). Prior to the study commencing, it was registered at the ClinicalTrials register (www.clinicaltrials.gov, identifier NCT02289872). With voluntary written, informed consent, 120 paramedics were recruited that satisfied the following inclusion criteria: (1) they had not performed more than 100 clinical adult (human) intubations by DL and no experience with clinical pediatric (human) intubation, and (2) they had not received any training in ETI using TruView device prior to the study. The study was conducted between November and December 2014.

### Simulation of the scenario

Each participant performed orotracheal intubations on a PediaSIM CPR training manikin (FCAE HealthCare, Sarasota, FL, USA). Subjects participated in three airway scenarios:

- The control scenario, in which neither chest compression nor cervical stabilization was applied during intubation.
- The chest compression scenario, in which continuous chest compression was applied using the LUCAS-2 chest compression system (Physio-Control, Redmond, WA, USA). Chest compression was provided at a rate of 100 min^−1^ to a depth of 5–6 cm during all intubation procedures.
- The chest compression with cervical stabilization scenario, in which both chest compression using Lucas-2 and cervical stabilization were applied. A correctly fitting standard cervical immobilization collar (StifNeck Select, Laerdal, Stavanger, Norway) was applied to the manikin’s neck to prevent movement of the cervical spine.

In each scenario, the manikin was placed in a neutral position on the floor of a well-lit room. The elevation of the head or the upper body was not allowed.

### Devices

**Fig. 1:**
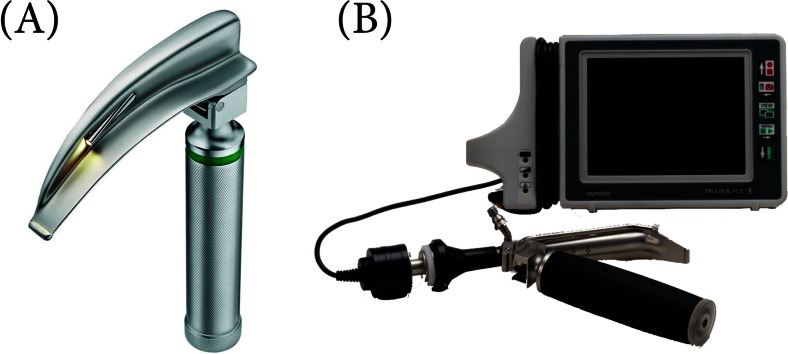
Laryngoscopes used for this study were **a** Macintosh Laryngoscope, **b** TruView PCD Videolaryngoscope

All the participants completed a 45-min training program prior the study, including an introduction to the anatomy and physiology of the airway and the techniques of ETI using a laryngoscope with Macintosh blade no. 2 (MAC; HEINE Optotechnik, Munich, Germany) and the TruView PCD video laryngoscope (TruView; Blade # 2, Truphatek Int.; Netanya, Israel) (Fig. 1). The Truview PCD video is intended to enable the medical professional to perform routine and difficult oral intubation cases while using a minimal amount of force and with a reduced rate of side effects to the patient, such as sore throat or soft tissue damage. TruView blade can be connected to a dedicated 5-in. LCD monitor via a unique camera for obtaining clear visual pictures of the intubation process. In this way, clinical safety is greatly improved and the incidence of incorrectly positioned endotracheal tube is reduced. The addition of oxygen during the intubation procedure via the unique oxygen port on the Truview PCD blades serves to slow the rate of desaturation, prevents the accumulation of mist and secretions on the lenses, and ensures a clear visual picture of the entire procedure [7, 18]. All intubations were performed using a tracheal tube with 5.0-mm internal diameter (ID). Lubricant was pre- applied to the tracheal tube, and a 10 mL syringe to block the tube’s cuff as well as an AMBU resuscitator bag (AMBU, Copenhagen, Denmark) were readily available and within range of the participants. After the training section, the participants were given 10 min to practice ETI with the three laryngoscopes.

### Study protocol

**Fig. 2:**
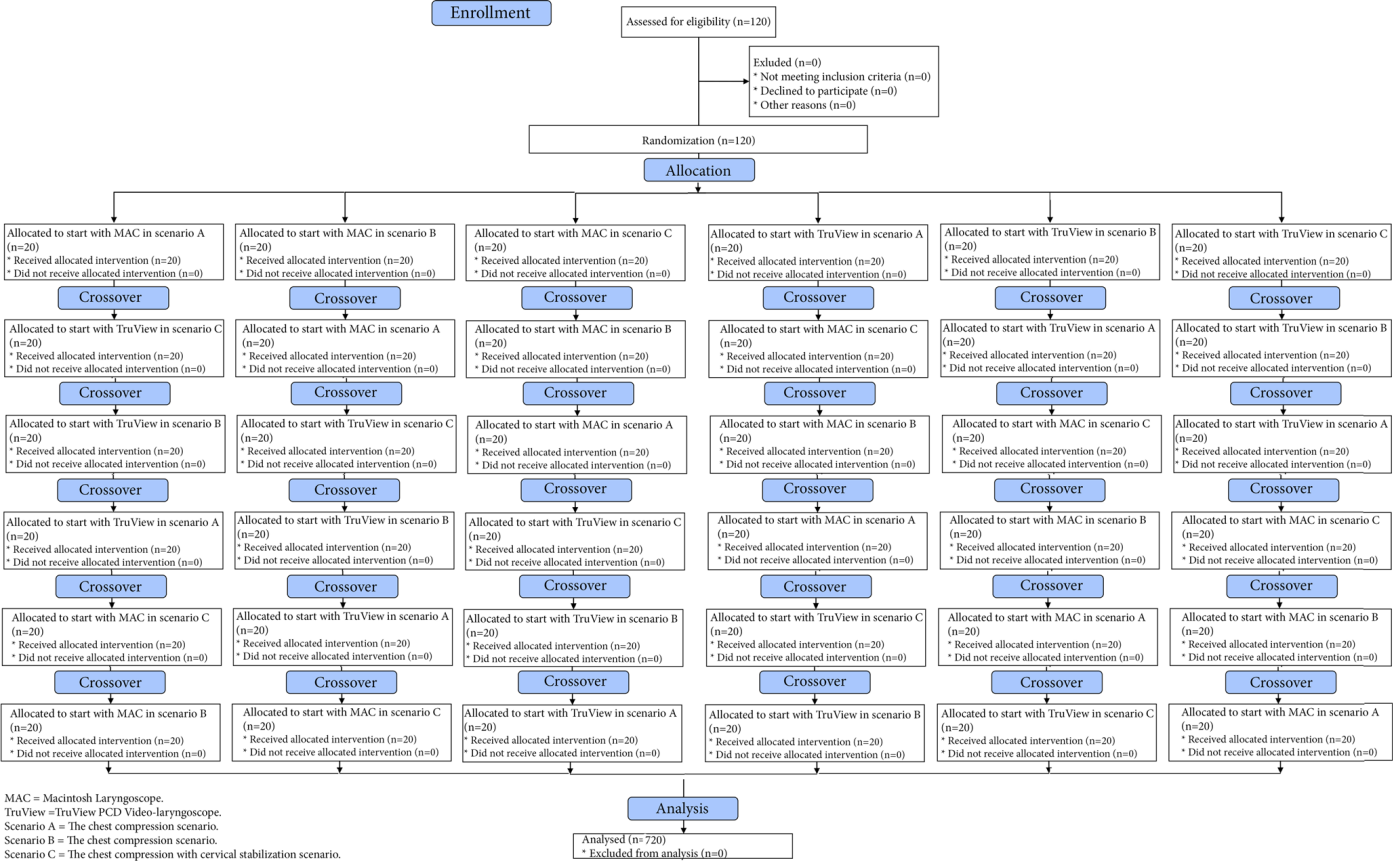
Flow chart of design and recruitment of participants according to CONSORT statement

A Research Randomizer program was used [www.researchrandomizer.com] to divide the participants into six groups and determine the order in which the different ETI devices were applied within each group. The first group attempted ETI using the MAC in scenario A, the second using the MAC in scenario B, the third using the MAC in scenario C, the fourth using the TruView in scenario A, the fifth using the TruView in scenario B, and the sixth using the TruView in scenario C (Fig. 2). After completing the ETI procedure, the participants had a 10 min break before performing intubation using another laryngoscope. The participants had a maximum of three attempts for ETI with each intubation method.

The participants were reminded before each attempt that the “patient” needs emergency ETI as quickly as possible to give them the feeling of time pressure that would be present in real emergency patients.

### Measurements and outcomes

The primary outcome of the study was time to intubation, defined as the time from insertion of the laryngoscope blade between the teeth to the first manual ventilation of the manikin’s lungs.

The secondary outcome was success of the intubation attempt (i.e., tracheal or oesophageal placement of the tube) which was recorded when the success of the ventilation attempt was confirmed by the manikin’s ventilation indicators. After each attempt, the participants were asked to rate the glottic view they had during the attempt using a Cormac and Lehane Grade [4]. The severity of the potential dental trauma was calculated based on a previously described [29] grading scale of the pressure on the teeth (0 = none, 1 = mild, 2 = moderate, and 3 = severe) by the same investigator. To access subjective opinions about the difficulty of the each intubation method, the participants were asked to give a rating on a visual analogue scale (VAS) with a score from 1 (extremely easy) to 10 (extremely difficult).

### Statistical analysis

Times needed to successful intubation were compared using the Wilcoxon signed-rank test. McNemar’s test was used to detect possible differences in success rates for ETI. For all statistical analysis, the R statistical package version 3.0.0 for Windows was used. *P* < 0.05 was considered as statistically significant. For comparisons of VAS, a one-way analysis of variance with a post hoc (Scheffé’s) test was used. Results are shown as means ± standard deviation (SD) or absolute numbers and percentages.

## Results

### Demographic testing

One hundred twenty paramedics (46 female, 38.3 %) participated in this study. No participant had previously performed a pediatric intubation with any laryngoscope. Eighty-seven participants (21 female, 24.1 %) worked in EMS teams, 33 participants (25 female, 75.5 %) worked in hospital emergency units. Mean age was 27.5 ± 5.8 years, and mean work experience was 3.7 ± 2.1 years.

### Scenario A: the control scenario

**Fig. 3:**
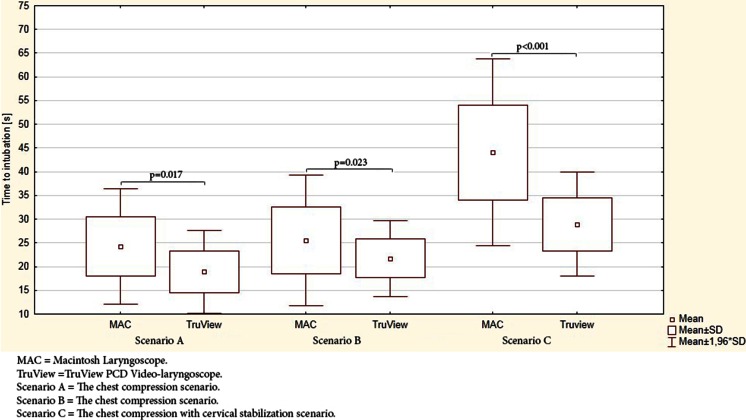
Comparison of time to intubation of the study devices in seconds

In the control scenario, overall effectiveness of intubation using the MAC and TruView was 100 %. However, the success rate after the first attempt using the MAC and TruView varied and amounted to 95.8 vs. 100 %, respectively. The average times to successful intubation using MAC and TruView are presented in Fig. 3. Time to intubation was achieved fastest with TruView (18.5 ± 4.5 s) and was significantly slower with MAC (24.3 ± 6.2 s, *p* = 0.017).

### Scenario B: the chest compression scenario

In the chest compression scenario, the difference in time to intubation between MAC and TruView was significant (25.7 ± 7.1 s vs. 21.6 ± 4.1 s; *p* = 0.023). The success rate after the first attempt using the distinct laryngoscopes varied and amounted to 57.5 vs. 100 % (MAC and TruView, respectively). There was a statistically significant difference between MAC and TruView in first intubation attempt effectiveness (*p* < 0.001) and overall effectiveness (*p* = 0.011).

### Scenario C: the chest compression with cervical stabilization scenario

In the chest compression with cervical stabilization scenario, time to intubation was significantly longer with the MAC (45.4 ± 10.3 s) than the TruView (28.9 s ± 5.6 s; *p* < 0.001). First-attempt success was achieved in 98.3 % of the TruView group compared with 45.8 % in the MAC group (*p* < 0.001). Overall effectiveness of TruView was 100 %, which was significantly higher than that of the MAC (68.4 %, *p* < 0.001) (Table 1).

**Table 1:** Intubation success for all scenarios

Success rate	Scenario A		Scenario B		Scenario C	
	MAC	TruView	MAC	TruView	MAC	TruView
First (%)	115 (95.8 %)	120 (100 %)	69 (57.5 %)	120 (100 %)	55 (45.8 %)	118 (98.3 %)
Second (%)	120 (100 %)	120 (100 %)	90 (75 %)	120 (100 %)	78 (65.0 %)	120 (100 %)
Third (%)	120 (100 %)	120 (100 %)	98 (81.7 %)	120 (100 %)	82 (68.3 %)	120 (100 %)
Failed (%)	0 (0.0 %)	0 (0.0 %)	22 (18.3 %)	0 (0.0 %)	38 (31.7 %)	0 (0.0 %)

*MAC* Macintosh Laryngoscope, *TruView* TruView PCD Video-laryngoscope. *Scenario A* The chest compression scenario, *Scenario B* The chest compression scenario, *Scenario C* The chest compression with cervical stabilization scenario

### Measures of difficulty in intubation

Glottic view quality was better with TruView than MAC in all scenarios, *p* < 0.001 (Table 2). Dental compression was also significantly lower with the TruView compared to MAC in all scenarios, *p* < 0.001 (Table 2). The ETI was most easily achieved with TruView compared to MAC in all scenarios: 2.4 vs. 2.6 points, for scenario A (*p* = 0.75); 2.7 vs. 3.9 points, for scenario B (*p* = 0.012); and 3.4 vs. 5.6 points, for scenario C (*p* < 0.001), respectively.

**Table 2:** Measures of difficulty in intubation

Parameter		Scenario A		Scenario B		Scenario C	
		MAC	TruView	MAC	TruView	MAC	TruView
Reported Cormack-Lehane grade	I	118 (98.3 %)	120 (100 %)	79 (65.8 %)	120 (100 %)	41 (34.2 %)	114 (95.0 %)
	II	2 (1.7 %)	0 (0.0 %)	27 (22.5 %)	0 (0.0 %)	56 (46.7 %)	6 (5.0 %)
	III	0 (0.0 %)	0 (0.0 %)	14 (11.7 %)	0 (0.0 %)	23 (19.2 %)	0 (0.0 %)
	IV	0 (0.0 %)	0 (0.0 %)	0 (0.0 %)	0 (0.0 %)	0 (0.0 %)	0 (0.0 %)
Dental compression scale	0	34 (28.3 %)	107 (89.2 %)	14 (11.7 %)	99 (82.5 %)	11 (9.2 %)	59 (49.2 %)
	1	56 (46.7 %)	13 (10.8 %)	69 (57.5 %)	21 (17.5 %)	41 (34.2 %)	49 (40.8 %)
	2	27 (22.5 %)	0 (0.0 %)	30 (25.0 %)	0 (0.0 %)	49 (40.8 %)	12 (10.0 %)
	3	3 (2.5 %)	0 (0.0 %)	7 (5.8 %)	0 (0.0 %)	19 (15.8 %)	0 (0.0 %)

*MAC* Macintosh Laryngoscope, *TruView* TruView PCD Video-laryngoscope, *Scenario A* The chest compression scenario, *Scenario B* The chest compression scenario, *Scenario C* The chest compression with cervical stabilization scenario

## Discussion

The DL using a laryngoscope with either a Miller or Macintosh blade is the main method of child intubation. The MAC is suitable for the treatment of children over 2 years [23, 37]. Therefore, a training minikin resembling a 6 years old was used in the study and intubation was performed with Macintosh blade. However, it should be noted that the effectiveness of intubation on children performed by paramedics in prehospital conditions using a laryngoscope with Miller or Macintosh blades is varied and ranges from 63.4 to 77 % [8, 9, 35, 38]. The problem of unsatisfactory efficacy of the first attempts of pediatric intubation applies not only to paramedics, but also to doctors who are not anesthesiologists [3]. Due to this, video laryngoscopy may be an alternative to DL, both for children and adults ETI [19, 28].

Following a single training session, the 120 paramedics recruited for our study had more pediatric intubation success with the TruView than the MAC. Previously, no study had compared TruView and MAC in pediatric intubations performed by paramedics in simulated chest compression scenarios or chest compression with cervical stabilization scenarios.

Chest compressions increased the time to intubation for both devices: a mean time of 3.4 s for MAC and 3.1 s for TruView. Other studies have also shown that time to intubation using DL increases when uninterrupted chest compressions are applied [13, 34, 39].

The overall effectiveness of intubation using the MAC in our study was 100 % for the control scenario, 81.7 % for the chest compression scenario, and 68.3 % for the chest compression with cervical stabilization scenario. Time to intubation in these scenarios varied and amounted to 22.3 vs. 25.7 s vs. 45.4 s, respectively. Mutlak et al. showed that effectiveness of the MAC for routine tracheal intubation in infants with normal airways was 100 %, and time to intubation was 26 s [20]. The study by Rodríguez-Núñez et al. [27] evaluating the intubation time using a Miller laryngoscope and GlideScope videolaryngoscope performed by 23 residents, indicated that the videolaryngoscope Glidescope® does not improve performance in this setting, and the time to intubation using Miller laryngoscope was 28.2 s (20.4–34.4). In our study with normal airway intubation, overall effectiveness of using the MAC was also 100 %. Nileshwar and Garg showed that the success ratio of orotracheal intubation in pediatric patients with simulated restriction of cervical spine movements using a short-handled MAC by anaesthesiologists was 100 % [21].

The mean intubation time using TruView during the chest compression scenario was 21.6 s, which was comparable to the results obtained in another study (20.1 s; IQR 18–23.3 s) [33]. Overall, the effectiveness of intubation using TruView was 100 % for the control scenario, the chest compression scenario, and the chest compression with cervical stabilization scenario. Time to intubation in these scenarios varied and amounted to 18.5 s vs. 21.6 s vs. 28.9 s, respectively. The success ratio of intubation during chest compression using TruView in study by Szarpak et al. was also 100 % [33]. In the study by Riveros et al. concerning patients (neonate up to 10 years of age) who were scheduled for general surgical procedures, times to intubation were 44 s and 23 s with the Truview PCD and DL, respectively [30]. In the case of TruView PCD, time to intubation was shorter in each scenario than in the study by Riveros et al. [26]. This may be due to the fact that intubation was performed on a minikin, not on a human.

In our study, the Cormack-Lehane graded views attained using the TruView PCD video laryngoscope were superior to the views attained using Macintosh laryngoscopy in all scenarios. The study by Riveros et al. showed no differences in Cormac-Lehane views between TruView and MAC [26]. Many studies have shown the superiority of video laryngoscopy over DL, especially in emergency intubation for both pediatric and adult patients [5, 6, 16, 25, 31].

Several limitations have to be noted. First, the procedures were performed on manikins, not on live subjects. Manikin studies can never fully replace studies on humans; however, the decision to use a standardized airway model was made intentionally as manikin studies allow researchers to simulate clinical practice conditions with strict standardization, thus allowing them to investigate thoroughly [24]. Several studies, on the other hand, have shown that the manikin used in considered to be the best manikin overall for the tasks performed in this study [15, 32]. Besides, these devices have not been compared in this situation in a randomized, controlled trial. Moreover, according to the International Liaison Committee on Resuscitation (ILCOR), randomized clinical trials for cases of cardiac arrest are unethical and cannot determine the expected benefits of CPR [17]. The second limitation is that we used inexperienced intubators; therefore, the results may have been less pronounced in more experienced hands. However, we believed that novice intubators would offer a more reliable comparison because they had little prior experience in pediatric intubation with either technique and would be less likely to display any bias. Although all participants prior the study received an 45-min standardized intubation training session. The strengths of this study include the use of a highly advanced patient simulator for performing pediatric advanced life support and the randomized crossover procedure.

The results from our study showing higher efficiency intubation using Truview PCD by paramedics show that short training is sufficient in order in this professional group to performed highly proficient with TruView PCD during intubation manikin. Further clinical studies are necessary to confirm these initial positive findings.

## Conclusions

The TruView offers better intubation conditions than the MAC on a pediatric manikin in all the scenarios test. The TruView may be used to elevate the epiglottis for orotracheal intubation. Further clinical studies are necessary to confirm these initial positive findings.

## Abbreviations

CPR: Cardiopulmonary resuscitation
DAS: Difficult airway society
DL: Direct laryngoscopy
ERC: European Resuscitation Council
EMS: Emergency Medical Service
ETI: Endotracheal intubation
GCS: Glasgow Coma Scale
MAC: Macintosh laryngoscope
SD: Standard deviation
PEEP: Positive end-expiratory pressure
VAS: Visual Analogue Scale
